# Smoking Exposure and the Risk of Latent Tuberculosis Infection: Results from NHANES 2011–2012

**DOI:** 10.3390/toxics12010094

**Published:** 2024-01-22

**Authors:** Xinsong Hu, Jiongya Liu, Yan Shao, Guoli Li, Honghuan Song, Qiao Liu, Cheng Chen, Limei Zhu

**Affiliations:** 1 School of Public Health, Nanjing Medical University, Nanjing 211166, China; huxinsong0808@163.com (X.H.); 17352923922@163.com (J.L.); 2 Department of Chronic Communicable Disease, Center for Disease Control and Prevention of Jiangsu Province, Nanjing 210009, China; maimai9947@163.com (Y.S.); liguoli_02468@163.com (G.L.); shh_0121@163.com (H.S.); liuqiaonjmu@163.com (Q.L.)

**Keywords:** tuberculosis, latent tuberculosis infection, smoking, NHANES, cotinine

## Abstract

The association between smoking exposure and latent tuberculosis infection (LTBI) has been investigated in a few studies; however, further investigation is needed. In this study, the 2011–2012 NHANES population was used to evaluate smoking exposure and LTBI risk. A total of 7042 participants with available LTBI results and without active tuberculosis were included for analysis. Smoking was defined as participants who smoked at least 100 cigarettes in their life. Both univariable and multivariable analysis were adopted to evaluate smoking exposure, as well as related factors on the risk of LTBI. LTBI rates among current smokers (12.1%) and former smokers (9.9%) were higher than non-smokers (5.9%). However, current smokers and former smokers were not significantly associated with LTBI risk when compared to non-smokers after adjusting by age and sex in the multivariable analysis. Meanwhile, we found that passive smoking was not associated with LTBI (adjusted odds ratio (AOR), 0.85; 95%CI, 0.66–1.09). In multivariable analysis, current smoking was associated with LTBI (OR, 1.67; 95%CI, 1.28–2.19), while former smokers had an increased OR of LTBI, but the OR did not reach statistical significance (OR, 1.15; 95%CI, 0.90–1.48). Household tuberculosis (TB) contact was also related to LTBI (OR, 1.93; 95%CI, 1.25–2.99). However, BMI and diabetes were not found to be associated with LTBI. Smoking, especially current smoking, was significantly associated with LTBI. LTBI screening should be recommended for active smokers. Former smoking and passive smoking exposure were not found to have a significant relationship with LTBI risk. However, the high LTBI rate among quitters indicated we should pay more attention to former smokers with LTBI.

## 1. Background

According to the WHO, tuberculosis (TB) is still one of the leading causes of human infectious diseases in the world; an estimated 10.6 million people fell ill with TB in 2021 [1]. Meanwhile, latent tuberculosis infection (LTBI) is a persistent immune response to *Mycobacterium* tuberculosis (MTB) infection without clinical symptoms or imaging features, and 5–10% of people with MTB infection will develop active TB. It was estimated that a quarter of the world’s population is infected with MTB [2]. Ambient air pollutants, such as tobacco smoking, biomass fuel, outdoor particles, etc., cause various kinds of human diseases. Air pollution has been reported to be associated with pneumonia-related morbidity and mortality in Europe [3]. Studies have shown that ambient air pollution, such as tobacco smoking and wildfire smoking, were associated with an increased risk of tuberculosis in several parts of the world [4,5]; a recent nationwide study conducted in China reported that long-term exposure to air pollution was related to the incidence of pulmonary tuberculosis [6].

A previous meta-analysis of 18 observational studies showed secondhand smoking (SHS) was not associated with LTBI [7], and this study concluded with high heterogeneity. Recent studies demonstrated that passive smoking reported an association with an increased risk of LTBI (mostly among children) [8,9], but these results may not be generalizable to the general population.

A former study used the National Health and Nutrition Examination Survey data in 1999, and revealed that smoking was associated with LTBI risk, but LTBI was only determined by the tuberculin skin test (TST) in this study [10]. A recent study employed the interferon-gamma release assay (IGRA) method for LTBI detection among special patients with respiratory diseases, and it revealed that smoking was associated with LTBI risk among a small sample size population [11].

Cigarette smoking increasing the likelihood of tuberculosis was reported recently [7]. Some theories supported the idea that ambient particles may impair the immunity of the human and would increase the risk of LTBI [12]. In this study, we want to know whether active and passive smoking were associated with LTBI. The National Health and Nutrition Examination Survey (NHANES) is a large, representative, population-based survey that provides estimates of disease prevalence in the United States [13]. Before 2011–2012, testing for LTBI in NHANES was most recently performed in 1999–2000, and the method of LTBI testing was the tuberculin skin test (TST), rather than the interferon-gamma release assay (IGRA) method. In 2011–2012, IGRA was first employed to evaluate the status of LTBI. Meanwhile, active and passive smoking (including indoor and outdoor smoking) were collected in the NHANES. Thus, we analyzed whether active or passive smoking were associated with LTBI risk among this large representative population.

## 2. Methods

### 2.1. Study Design and Population

NHANES is a cross-sectional study in the United States which can select representative samples of the non-institutionalized national population. The data came from interview, health examination, and laboratory measurements. The goal of the study was to refine which sub-populations of smoking could benefit from the LTBI screening. We included all the participants with available results of QuantiFERON-TB Gold in-Tube (QFT). Of the 7144 NHANES 2011–2012 participants with QFT results, 63 had indeterminate or missing QFT results, and 39 were active tuberculosis patients. Thus, 7042 subjects were finally included in the analyses.

### 2.2. Measures and Definitions

QFT results were interpreted by the guidelines from the Centers for Disease Control and Prevention for using interferon-gamma release assays (IGRAs). The QFT result of no less than 0.35 IU/mL was defined as LTBI-positive; participants with less than 0.35 IU/mL were defined as LTBI-negative. The population was classified as LTBI-negative and LTBI-positive, after excluding indeterminate and missing participants. Smoking was determined as having smoked at least 100 cigarettes in their life. A current smoker was defined as an adult who had smoked 100 cigarettes in his or her lifetime and who currently smoked cigarettes. Former smoker was defined as a participant who had smoked at least 100 cigarettes in his or her lifetime but had quit smoking at the time of the interview. A non-smoker was defined as never having smoked or smoked less than 100 cigarettes in their lifetime [14]. Meanwhile, cotinine was used as a biomarker to indicate whether the individual had been exposed to nicotine recently. Cotinine was categorized into three levels according to its association with smoking exposure. Cotinine level < 1 ng/mL represented no smoking exposure. If the individual was exposed to typical amounts of secondhand smoke, the cotinine level could increase to 1–10 ng/mL. If the individual was heavily exposed to smoking, the cotinine level was almost always >10 ng/mL [15]. Diabetes status was determined by HbA1c, and the classification of diabetes was defined according to the American Diabetes Association (ADA) [16]. HbA1c less than 5.7% was considered as normal, HbA1c no less than 5.7% and less than 6.5% was considered as pre-diabetes, and HbA1c no less than 6.5% was defined as diabetes. Body mass index (BMI) was defined according to WHO guidelines [17]. Briefly, BMI was categorized into underweight (less than 18.5), healthy weight (between 18.5 and 25), overweight (between 25.0 and 30), and obese (30.0 or more).

### 2.3. Data Analysis

In the original NHANES data, responses of “don’t know”, “refused”, and “missing” were regarded as missing and were not shown in the tables. χ^2^ test or Fisher exact tests were used to compare the categorical data where applicable. Odds ratios (OR) and 95% confidence intervals (CI) were calculated for univariable and multivariable logistic regression. The ORs for risk factors of LTBI were adjusted by age and sex. All analyses were conducted using SAS 9.3 software (SAS Institute, Inc., Cary, NC, USA). The level of statistical significance was set at *p* < 0.05.

## 3. Results

### Study Participants

By excluding indeterminate QFT results and active tuberculosis in the baseline population, a total of 7042 participants were included for analysis. The mean age was 37 years (interquartile range (IQR), 17–56). Males accounted for 49.8% of participants. The majority of people were born in the US (75%), and the non-Hispanic White population accounted for the highest percentage (33.1%) (Table 1).

As shown in Table 2, the older age groups were significantly associated with LTBI when compared to the age group less than 20 years; the age group no less than 60 years had a higher OR of LTBI with an LTBI rate of 15.4% (odds ratio, [OR], 13.6; 95%CI, 9.14–20.24) when compared to the age group less than 20 years old. Females had a relatively lower LTBI rate compared to males (6.3% vs. 8.6%), and female status was significantly associated with a lower odds ratio of having LTBI (OR, 0.71; 95%CI, 0.59–0.85). People who were underweight had the lowest LTBI rate (2.4%); the other BMI groups were not found to be significantly associated with LTBI compared to the underweight group. People not born in the US had a higher LTBI rate (18.8%) than those who were born in the US (3.6%); the OR of LTBI increased by 4.84-fold (adjusted odds ratio (AOR), 5.84; 95%CI, 4.81–7.08). When comparing LTBI rates among different races, we found that non-Hispanic Black, Mexican American, other Hispanic, and other races had significantly higher LTBI rates compared to the non-Hispanic White population (*p* < 0.0001 for all comparisons).

Even though the LTBI rate among current smokers (12.1%) and former smokers (9.9%) was higher than non-smokers (5.9%), current smokers and former smokers were not found to be significantly associated with LTBI when compared to non-smokers after adjusting by age and sex. The cotinine level generally demonstrated the degree of smoking [18], so we classified those with cotinine lower than 1 ng/mL as no smoking exposure, and those between 1–10 ng/mL as secondhand smoke exposure, and those with cotinine no less than 10 ng/mL as heavy smoking exposure. However, compared to no smoking exposure, people under secondhand smoke exposure had no significant association with LTBI (AOR, 0.93; 95%CI, 0.56–1.52), and heavy smoking based on cotinine level were not found in association with LTBI (AOR, 0.99; 95%CI, 0.79–1.25). Meanwhile, we found that passive smoking was not associated with LTBI (AOR, 0.85; 95%CI, 0.66–1.09). Household TB contact was significantly associated with LTBI (AOR, 1.86; 95%CI, 1.23–2.81). Meanwhile, diabetes was found to be associated with LTBI (AOR, 1.43; 95%CI, 1.11–1.84).

We included age, sex, BMI, country of birth, smoking status, household TB contact history, and diabetes in multivariable analysis. Increasing age was significantly associated with LTBI (OR, 1.03; 95%CI, 1.03–1.04), while the female sex was significantly associated LTBI with an OR of 0.75 (OR, 0.75; 95%CI, 0.61–0.92), which indicated that the female sex would be a protective factor in LTBI; people born outside the US, and current smoking were significantly associated with LTBI (OR, 6.22; 95%CI, 5.06–7.65; OR, 1.67; 95%CI, 1.28–2.19). Household TB contact was significantly related to LTBI (OR, 1.93; 95%CI, 1.25–2.99). However, BMI and diabetes were not found in significant association with LTBI (Table 3).

We further investigated the relationship between cotinine levels and smoking status. As shown in Table 4, a large proportion of people quit smoking (16.0%, 1105/6898). Meanwhile, most of the quitters (84.4%, 933/1105) had a cotinine level of less than 1 ng/mL. However, the LTBI rate among quitters was highest (12.1%).

## 4. Discussion

In this cross-sectional study with a large sample size, LTBI status was determined by the IGRA method. We found current smoking was independently associated with LTBI. However, quitting smoking was not found to be significantly related to LTBI even though the quitters had a much higher LTBI rate. Passive smoking, not only passive smoking at home, but also passive smoking at the workplace, was not significantly associated with LTBI. Meanwhile, the cotinine level was not found to be associated with LTBI. However, old age, the male sex, birth outside of the US, and household TB contact were independently associated with LTBI in this study.

Smoking may impair human pulmonary immunity to *Mycobacterium* tuberculosis [19]. A study conducted by Horne et al. revealed that smoking was associated with an increased risk of LTBI, especially for current smokers [10]. In that study, LTBI was determined by TST, which might overestimate the rate of LTBI. Compared to TST, IGRA was employed in this study to confirm the status of LTBI, and we also found that smoking was associated with LTBI, especially for current smokers. Thus, without considering the magnitude of the relationship between smoking and LTBI, either the TST or IGRA method was capable of revealing the positive relationship between smoking on LTBI.

Smoking was found to be associated with LTBI, but quitting smoking seemed not to revert LTBI status. Studies have proven that smoking not only increased the risk of TB, but also increased the risk of recurrent TB and impaired the response to TB treatment [20,21]. Thus, cessation of smoking would decrease the risk of developing TB. However, the LTBI rate among former smokers was higher than those of current smokers. We further analyzed the data and found the age of the former smokers was higher than current smokers (57.3 vs. 44.8 years), which may explain why quitters had a higher LTBI rate than the current smokers. However, the OR on LTBI reached no significance when compared to the non-smokers.

Cotinine was not significantly related to LTBI. However, quitters with a lower cotinine level still had a high LTBI rate, compared to those smokers who had a cotinine level higher than 10 ng/mL. LTBI status seemed not to be reverted by cessation of smoking, which means cessation of smoking would not help the reversion of LTBI. Previous studies did not find that cotinine was related with LTBI [10].

Secondhand smoking may induce inflammation and impair immunity to respiratory infections [22]. However, passive smoking in the workplace was not associated with LTBI in our population-based study; passive smoking at home only marginally increased the OR on LTBI. A previous study indicated that passive smoking increased the risk of LTBI, but still reached no statistical significance [23].

In the univariable analysis of the relationship between diabetes and LTBI, there was a positive relationship between diabetes and LTBI. However, in the multivariable analysis, diabetes was not associated with LTBI. We found the age of the diabetes population was higher than the normal-glucose people (59.7 vs. 39.7 years). Some studies have suggested that diabetes may increase the risk of acquiring a latent tuberculosis infection, and it might also be associated with an increased risk of progressing from latent tuberculosis to active TB disease. Diabetes could impair immune function, making individuals more susceptible to infections, including tuberculosis [24]. A systematic review indicated that the cohort study revealed an increased but nonsignificant risk of LTBI among diabetics, but, for the cross-sectional studies, they reached a small and marginally significant relationship between diabetes and LTBI risk [25]. A recent meta-analysis showed a positive association between diabetes and LTBI risk from cohort and cross-sectional studies [26]. The relationship between diabetes and latent tuberculosis infection may be influenced by various factors such as glycemic control, the duration of diabetes, and the presence of other comorbidities. Thus, the strategy of employing mass screening of LTBI among the diabetes population is still an ongoing issue for research.

This study had some limitations. First, NHANES is a cross-sectional study, so the causality relationship between smoking exposure and LTBI risk would not be established, and a prospective study would be better to explain the causality. Second, the LTBI status was determined by the cut-off value of the IGRA method. The borderline effect of the cut-off value would improperly classify the status of LTBI, but the IGRA method would be better in order to have a higher specificity than the skin tuberculin test, until now. Third, the information on smoking was self-reported, so the recalling bias would not be excluded, especially for the former smokers. However, we used the cotinine level as a control to show the smoking status: 89.9% of non-smokers had cotinine less than 1 ng/mL, and 93.3% of smokers had a cotinine level no less than 10 ng/mL.

In conclusion, our results implied that LTBI screening should be considered for active smokers. Even though cessation of smoking was not found to be associated with LTBI risk, the high LTBI rate among the quitters suggests we should pay more attention to these former smokers with LTBI.

## Figures and Tables

**Table 1:** Characteristics information of the enrolled populations.

Characteristic	N = 7042	%
Age (years)		
<20	2117	30.1
≥20 and <40	1751	24.9
≥40 and <60	1630	23.1
≥60	1544	21.9
Sex		
Male	3509	49.8
Female	3533	50.2
BMI		
<18.5	827	11.7
≥18.5 and <25	2323	33.0
≥25.0 and <30	1856	26.4
≥30	1953	27.7
Country of birth		
US	5279	75.0
Others	1760	25.0
Race		
Mexican American	894	12.7
Other Hispanic	739	10.5
Non-Hispanic White	2331	33.1
Non-Hispanic Black	1906	27.1
Other race—including multi-racial	1172	16.6
Smoking		
Never	4936	70.1
Current smoker	985	14.0
Every day	811	11.5
Some times	174	2.5
Quitter	1121	15.9
Cotinine (ng/mL)		
<1	5272	74.9
≥1 and <10	372	5.3
≥10	1254	17.8
Passive smoking in the workplace		
Yes	398	5.7
No	2392	34.0
Passive smoking inside home		
Yes	945	13.4
No	6078	86.3
Passive smoking		
No	5779	82.1
Yes	1251	17.8
Household TB contact		
No	6802	96.6
Yes	196	2.8
Diabetes status		
Normal	3858	54.8
Pre-diabetes	1363	19.4
Diabetes	796	11.3

**Table 2:** LTBI status in association with the characteristics of the population.

Characteristic	LTBI+ (N = 523)	%	LTBI− (N = 6519)	%	AOR	95% CI		*p*
Age (years)								
<20	28	1.3%	2089	98.7%	1.00			
≥20 and <40	87	5.0%	1664	95.0%	3.90	2.54	6.00	<0.001
≥40 and <60	170	10.4%	1460	89.6%	8.69	5.79	13.03	<0.001
≥60	238	15.4%	1306	84.6%	13.60	9.14	20.24	<0.001
Sex								
Male	302	8.6%	3207	91.4%	1.00			
Female	221	6.3%	3312	93.7%	0.71	0.59	0.85	<0.001
BMI								
<18.5	20	2.4%	807	97.6%	1.00			
≥18.5 and <25	162	7.0%	2161	93.0%	1.32	0.81	2.16	0.267
≥25.0 and <30	171	9.2%	1685	90.8%	1.26	0.77	2.08	0.362
≥30	166	8.5%	1787	91.5%	1.19	0.72	1.95	0.500
Country of birth								
US	331	3.6%	1429	96.4%	1.00			
Others	523	18.8%	6516	81.2%	5.84	4.81	7.08	<0.001
Race								
Non-Hispanic White	76	3.3%	2255	96.7%	1.00			
Non-Hispanic Black	123	6.5%	1783	93.5%	2.91	2.15	3.93	<0.001
Mexican American	80	8.9%	814	91.1%	6.01	4.25	8.48	<0.001
Other Hispanic	93	12.6%	646	87.4%	6.17	4.44	8.58	<0.001
Other race—including multi-racial	151	12.9%	1021	87.1%	7.33	5.42	9.91	<0.001
Smoking								
Never	289	5.9%	4647	94.1%	1.00			
Current smoker	98	9.9%	887	90.1%	1.03	0.80	1.33	0.796
Quitter	136	12.1%	985	87.9%	0.98	0.77	1.23	0.833
Cotinine (ng/mL)								
<1	392	7.4%	4880	92.6%	1.00			
≥1 and <10	18	4.8%	354	95.2%	0.93	0.56	1.52	0.764
≥10	104	8.3%	1150	91.7%	0.99	0.79	1.25	0.947
Passive smoking in workplace								
No	198	8.3%	2194	91.7%	1.00			
Yes	34	8.5%	364	91.5%	1.03	0.70	1.54	0.865
Passive smoking inside home								
No	467	7.7%	5611	92.3%	1.00			
Yes	56	5.9%	889	94.1%	0.77	0.57	1.03	0.077
Passive smoking								
No	442	7.6%	5337	92.4%	1.00			
Yes	81	6.5%	1170	93.5%	0.85	0.66	1.09	0.205
Household TB contact								
No	488	7.2%	6314	92.8%	1.00			
Yes	30	15.3%	166	84.7%	1.86	1.23	2.81	0.003
Diabetes status								
Normal	246	6.4%	3612	93.6%	1.00			
Pre-diabetes	144	10.6%	1219	89.4%	1.10	0.87	1.38	0.434
Diabetes	124	15.6%	672	84.4%	1.43	1.11	1.84	0.005

Footnotes: AORs were adjusted by age and sex (except age and sex themselves).

**Table 3:** Multivariable analysis for factors related to LTBI.

Variables	OR	95%CI		*p*
Age (years)	1.04	1.03	1.04	<0.001
Sex				
0 = Male	Reference			
1 = Female	0.75	0.61	0.92	0.005
BMI				
0 = (<18.5)	Reference			
1 = (≥18.5 and <25)	0.70	0.38	1.29	0.252
2 = (≥25.0 and <30)	0.70	0.38	1.28	0.250
3 = (≥30)	0.78	0.42	1.43	0.415
Country of birth				
0 = US	Reference			
1 = Others	6.22	5.06	7.65	<0.001
Smoking				
0 = Never	Reference			
1 = Current smoker	1.67	1.28	2.19	<0.001
2 = Quitter	1.15	0.90	1.48	0.265
Household TB contact				
0 = No	Reference			
1 = Yes	1.93	1.25	2.99	0.003
Diabetes status				
0 = Normal	Reference			
1 = Pre-diabetes	0.98	0.77	1.25	0.868
2 = Diabetes	1.24	0.94	1.63	0.126

**Table 4:** The crosstab of cotinine level and self-reported smoking for LTBI status.

Cotinine (ng/mL)	Never Smoking n (LTBI%)	Smoker n (LTBI%)	Quitter n (LTBI%)	Total
<1	4313 (6.2%)	26 (11.5%)	933 (13.1%)	5272 (7.4%)
≥1 and <10	277 (2.2%)	39 (17.9%)	56 (8.9%)	372 (4.8%)
≥10	239 (4.6%)	899 (9.6%)	116 (6.0%)	1254 (8.3%)
Total	4829 (5.9%)	964 (10.0%)	1105 (12.1%)	6898 (7.5%)

For the quitters, they had a much higher LTBI than never smoking and current smokers. Even if they stopped smoking, the risk of LTBI seemed not to be reverted.

## Data Availability

All the data and material were shown in the manuscript.

## References

[1] Bagcchi S. (2023). WHO’s Global Tuberculosis Report 2022. Lancet Microbe.

[2] Houben R.M., Dodd P.J. (2016). The Global Burden of Latent Tuberculosis Infection: A Re-estimation Using Mathematical Modelling. PLoS Med..

[3] Liu S., Lim Y.H., Chen J., Strak M., Wolf K., Weinmayr G., Rodopolou S., de Hoogh K., Bellander T., Brandt J. (2022). Long-term Air Pollution Exposure and Pneumonia-related Mortality in a Large Pooled European Cohort. Am. J. Respir. Crit. Care Med..

[4] Linde L.R., Readhead A., Barry P.M., Balmes J.R., Lewnard J.A. (2023). Tuberculosis Diagnoses Following Wildfire Smoke Exposure in California. Am. J. Respir. Crit. Care Med..

[5] Lin H.H., Ezzati M., Murray M. (2007). Tobacco smoke, indoor air pollution and tuberculosis: A systematic review and meta-analysis. PLoS Med..

[6] Zhu S., Wu Y., Wang Q., Gao L., Chen L., Zeng F., Yang P., Gao Y., Yang J. (2022). Long-term exposure to ambient air pollution and greenness in relation to pulmonary tuberculosis in China: A nationwide modelling study. Environ. Res..

[7] Patra J., Bhatia M., Suraweera W., Morris S.K., Patra C., Gupta P.C., Jha P. (2015). Exposure to second-hand smoke and the risk of tuberculosis in children and adults: A systematic review and meta-analysis of 18 observational studies. PLoS Med..

[8] Blount R.J., Phan H., Trinh T., Dang H., Merrifield C., Zavala M., Zabner J., Comellas A.P., Stapleton E.M., Segal M.R. (2021). Indoor Air Pollution and Susceptibility to Tuberculosis Infection in Urban Vietnamese Children. Am. J. Respir. Crit. Care Med..

[9] Ganmaa D., Khudyakov P., Buyanjargal U., Jargalsaikhan B., Baigal D., Munkhjargal O., Yansan N., Bolormaa S., Lkhagvasuren E., Sempos C.T. (2019). Prevalence and Determinants of QuantiFERON-Diagnosed Tuberculosis Infection in 9810 Mongolian Schoolchildren. Clin. Infect. Dis..

[10] Horne D.J., Campo M., Ortiz J.R., Oren E., Arentz M., Crothers K., Narita M. (2012). Association between smoking and latent tuberculosis in the U.S. population: An analysis of the National Health and Nutrition Examination Survey. PLoS ONE.

[11] Alipour Fayez E., Moosavi SA J., Kouranifar S., Delbandi A.A., Teimourian S., Khoshmirsafa M., Reza Bolouri M., Sadeghi Shermeh A., Shekarabi M. (2020). The effect of smoking on latent tuberculosis infection susceptibility in high risk individuals in Iran. J. Immunoass. Immunochem..

[12] Torres M., Carranza C., Sarkar S., Gonzalez Y., Vargas A.O., Black K., Meng Q., Quintana-Belmares R., Hernandez M., Angeles Garcia J.J.F. (2019). Urban airborne particle exposure impairs human lung and blood *Mycobacterium tuberculosis* immunity. Thorax.

[13] Centers for Disease Control and Prevention, National Center for Health Statistics (NCHS) (2012). National Health and Nutrition Examination Survey Data.

[14] Chaiton M., Diemert L., Cohen J.E., Bondy S.J., Selby P., Philipneri A., Schwartz R. (2016). Estimating the number of quit attempts it takes to quit smoking successfully in a longitudinal cohort of smokers. BMJ Open.

[15] Pirkle J.L., Flegal K.M., Bernert J.T., Brody D.J., Etzel R.A., Maurer K.R. (1996). Exposure of the US population to environmental tobacco smoke: The Third National Health and Nutrition Examination Survey 1988 to 1991. JAMA.

[16] American Diabetes Association (2019). Classification and Diagnosis of Diabetes: Standards of Medical Care in Diabetes—2019. Diabetes Care.

[17] WHO Expert Consultation (2004). Appropriate body-mass index for Asian populations and its implications for policy and intervention strategies. Lancet.

[18] Parker D.R., Lasater T.M., Windsor R., Wilkins J., Upegui D.I., Heimdal J. (2002). The accuracy of self-reported smoking status assessed by cotinine test strips. Nicotine Tob. Res..

[19] O’Leary S.M., Coleman M.M., Chew W.M., Morrow C., McLaughlin A.M., Gleeson L.E., O’Sullivan M.P., Keane J. (2014). Cigarette smoking impairs human pulmonary immunity to Mycobacterium tuberculosis. Am. J. Respir. Crit. Care Med..

[20] Yen Y.F., Yen M.Y., Lin Y.S., Lin Y.P., Shih H.C., Li L.H., Chou P., Deng C.Y. (2014). Smoking increases risk of recurrence after successful anti-tuberculosis treatment: A population-based study. Int. J. Tuberc. Lung Dis..

[21] Bates M.N., Khalakdina A., Pai M., Chang L., Lessa F., Smith K.R. (2007). Risk of tuberculosis from exposure to tobacco smoke: A systematic review and meta-analysis. Arch. Intern. Med..

[22] Bhat T.A., Kalathil S.G., Bogner P.N., Miller A., Lehmann P.V., Thatcher T.H., Phipps R.P., Sime P.J., Thanavala Y. (2018). Secondhand Smoke Induces Inflammation and Impairs Immunity to Respiratory Infections. J. Immunol..

[23] Lindsay R.P., Shin S.S., Garfein R.S., Rusch M.L., Novotny T.E. (2014). The Association between active and passive smoking and latent tuberculosis infection in adults and children in the united states: Results from NHANES. PLoS ONE.

[24] Kumar N.P., Babu S. (2023). Impact of diabetes mellitus on immunity to latent tuberculosis infection. Front. Clin. Diabetes Healthc..

[25] Lee M.R., Huang Y.P., Kuo Y.T., Luo C.H., Shih Y.J., Shu C.C., Wang J.Y., Ko J.C., Yu C.J., Lin H.H. (2017). Diabetes Mellitus and Latent Tuberculosis Infection: A Systematic Review and Metaanalysis. Clin. Infect. Dis..

[26] Liu Q., Yan W., Liu R., Bo E., Liu J., Liu M. (2022). The Association Between Diabetes Mellitus and the Risk of Latent Tuberculosis Infection: A Systematic Review and Meta-Analysis. Front. Med..

